# The Overlooked Role of Psychiatrists in Obesity Management: A Multidisciplinary Perspective

**DOI:** 10.1192/j.eurpsy.2026.11376

**Published:** 2026-07-31

**Authors:** D. Dos Santos, A. Castanheira da Silva, F. Miranda

**Affiliations:** Psychiatry and Mental Health Department, Hospital Garcia de Orta, Almada, Portugal

## Abstract

**Introduction:**

Obesity is a chronic, multifactorial condition often associated with psychiatric comorbidities and a significant stigma burden. The role of the psychiatrist in obesity management has evolved but remains underutilized in multidisciplinary clinical practice.

**Objectives:**

To critically review the role of psychiatrists in obesity management, identify innovative approaches, and discuss limitations in the integration of psychiatry within multidisciplinary teams.

**Methods:**

Narrative review of recent literature on psychiatric interventions in obesity, analyzing guidelines, clinical trials, and experiences of multidisciplinary integration.

**Results:**

The inclusion of psychiatrists into multidisciplinary teams is crucial for addressing the behavioral, neurobiological, and psychosocial factors of obesity, particularly in bariatric surgery contexts.

New pharmacotherapies, such as tirzepatide and cagrilintide-semaglutide, show potential for weight management in patients with severe mental illness, but translation to psychiatric populations still requires robust studies.

Psychotherapeutic interventions, including cognitive-behavioral therapy, show benefits for psychiatric symptoms, but the impact on weight loss is modest and clinically limited. Third-wave approaches, such as acceptance and commitment therapy, have shown promising results in emotional management and treatment adherence.

Barriers to effective integration of psychiatrists persist, including a lack of training in metabolic health, absence of standardized protocols for screening and metabolic monitoring, and a scarcity of pragmatic clinical trials reflecting the complexity of real-world patients.

The dual stigma (obesity and mental illness) complicates access to and adherence to treatment, necessitating empathetic communication and personalized strategies.

**Conclusions:**

Despite advances in pharmacotherapy and psychotherapy, the psychiatrist’s role in obesity remains limited by a lack of formal integration, insufficient metabolic training, and restricted clinical evidence for combined interventions. Innovation lies in adopting collaborative models, interdisciplinary training, and developing clinical trials that include complex psychiatric populations. The psychiatrist should be a central agent in promoting integrated care, not only as a “gatekeeper” but as the longitudinal manager of metabolic risk and mental health.

**Disclosure of Interest:**

None Declared

